# Maturation of the Na,K-ATPase in the Endoplasmic Reticulum in Health and Disease

**DOI:** 10.1007/s00232-021-00184-z

**Published:** 2021-06-10

**Authors:** Vitalii Kryvenko, Olga Vagin, Laura A. Dada, Jacob I. Sznajder, István Vadász

**Affiliations:** 1 Department of Internal Medicine, Universities of Giessen and Marburg Lung Center (UGMLC), Member of the German Center for Lung Research (DZL), Justus Liebig University, Klinikstrasse 33, 35392 Giessen, Germany; 2 The Cardio-Pulmonary Institute (CPI), Giessen, Germany; 3 Department of Physiology, David Geffen School of Medicine, University of California at Los Angeles, Los Angeles, CA USA; 4 Veterans Administration Greater Los Angeles Healthcare System, Los Angeles, CA USA; 5 Division of Pulmonary and Critical Care Medicine, Feinberg School of Medicine, Northwestern University, Chicago, IL USA

**Keywords:** Na,K-ATPase, Endoplasmic reticulum, Protein maturation, Protein folding, Unfolded protein response

## Abstract

**Abstract:**

The Na,K-ATPase establishes the electrochemical gradient of cells by driving an active exchange of Na^+^ and K^+^ ions while consuming ATP. The minimal functional transporter consists of a catalytic α-subunit and a β-subunit with chaperon activity. The Na,K-ATPase also functions as a cell adhesion molecule and participates in various intracellular signaling pathways. The maturation and trafficking of the Na,K-ATPase include co- and post-translational processing of the enzyme in the endoplasmic reticulum (ER) and the Golgi apparatus and subsequent delivery to the plasma membrane (PM). The ER folding of the enzyme is considered as the rate-limiting step in the membrane delivery of the protein. It has been demonstrated that only assembled Na,K-ATPase α:β-complexes may exit the organelle, whereas unassembled, misfolded or unfolded subunits are retained in the ER and are subsequently degraded. Loss of function of the Na,K-ATPase has been associated with lung, heart, kidney and neurological disorders. Recently, it has been shown that ER dysfunction, in particular, alterations in the homeostasis of the organelle, as well as impaired ER-resident chaperone activity may impede folding of Na,K-ATPase subunits, thus decreasing the abundance and function of the enzyme at the PM. Here, we summarize our current understanding on maturation and subsequent processing of the Na,K-ATPase in the ER under physiological and pathophysiological conditions.

**Graphic Abstract:**

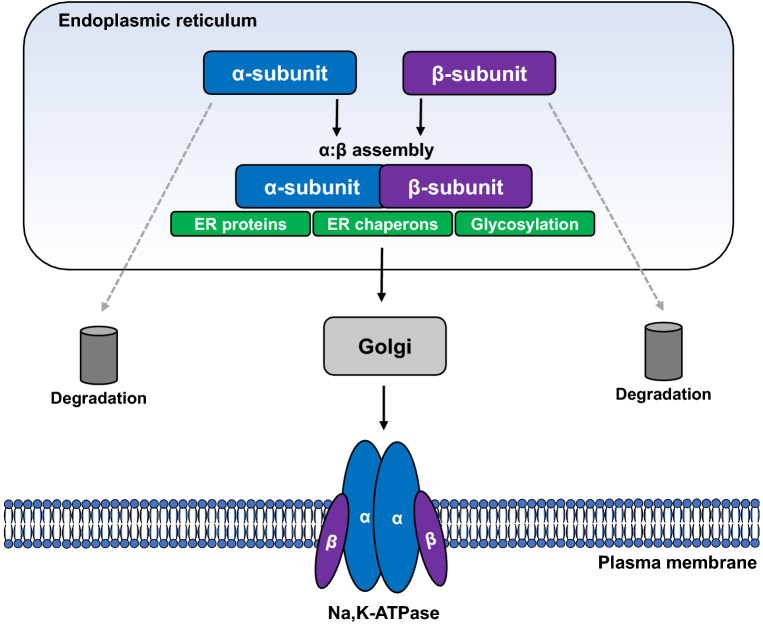

## Function, Structure and Regulation of the Na,K-ATPase

The Na,K-ATPase that is widely expressed in various tissues and organs is a heterodimeric enzyme and a member of the P-type ATPase family (Jorgensen et al. 2003; Kaplan 2002). In polarized cells, the Na,K-ATPase is localized at the basolateral membrane, where the transporter performs its primary function, establishment of a Na^+^/K^+^ gradient, which is achieved by pumping three Na^+^ ions out of the cell in exchange to two K^+^ ions while hydrolyzing a single ATP molecule. This activity of the Na,K-ATPase consumes up to 50–70% of total cellular ATP (Wieser and Krumschnabel 2001). The above-mentioned ion gradients are not only important for movement of Na^+^ and K^+^ across the cell, but also play an essential role in secondary active transport of ions (H^+^, Ca^2+^, Cl^−^), amino acids, sugars and neurotransmitters (Jorgensen et al. 2003; Kaplan 2002). Furthermore, ion gradients generated by the Na,K-ATPase are essential to control cell volume and to restore the resting membrane potential in excitable cells (Lingrel 2010; Yordy and Bowen 1993). In addition to ion transport, the Na,K-ATPase also acts as a cell adhesion molecule in polarized epithelial cells (Cereijido et al. 2012; Vagin et al. 2012), indirectly regulates permeability of tight junctions by leading to hyper-phosphorylation of occludin (Rajasekaran et al. 2007), controls the actin cytoskeleton, cellular volume and motility (Rajasekaran et al. 2001a, b). Moreover, the Na,K-ATPase participates in various intracellular signaling pathways by indirectly regulating Ca^2+^ concentrations (Tian and Xie 2008) and responds to oxidative stress (Figtree et al. 2012; Huang et al. 1994; Liu et al. 2012; Pratt et al. 2018).

The minimal functional Na,K-ATPase molecule consists of a catalytic α- and a N-glycosylated β-subunit.(Morth et al. 2009). As reviewed recently, four isoforms of the α-subunits (α_1_, α_2_, α_3_ and α_4_) and three isoforms of the β-subunits (β_1_, β_2_ and β_3_) have been identified, of which the α_1_:β_1_ combination is the most abundant. Of note, the heterodimeric composition of different α- and β-subunit isoforms is tissue-specific and modulates the kinetic properties and ion affinity of the transporter (Clausen et al. 2017).

Structurally, the Na,K-ATPase α-subunit has a molecular mass of ~ 110 kDa and consists of a large intracellular domain, ten transmembrane segments, and an extracellular domain. In contrast to the β-subunit, the α-subunit is tightly linked to the cellular cytoskeleton (Kaplan 2002). The α-subunit consists of three cytoplasmic domains [A (actuator), N (nucleotide binding) and P (phosphorylation)], which are required for ATP hydrolysis, and 10 transmembrane helices, where the binding sites for Na^+^ and K^+^ are localized (Kanai et al. 2013; Nyblom et al. 2013). Several phosphorylation sites in the α-subunit, e.g., Tyr^10^, Ser^16^, Ser^18^, Ser^23^ on the N terminus and Ser^943^ on the C terminus, regulate activity and internalization of the enzyme by intracellular signaling pathways (Aperia 2007; Feschenko and Sweadner 1995; Soltoff et al. 2010; Sweadner and Feschenko 2001).

The Na,K-ATPase β-subunit is a type II integral membrane protein. Depending on the glycosylation profile, it has a molecular weight of ~ 35–55 kDa and consists of a short N-terminal cytoplasmic, a single transmembrane and a large extracellular domain with three N-glycosylation sites (Tokhtaeva et al. 2011; Vagin et al. 2012). The main function of the Na,K-ATPase β-subunit is to serve as a chaperone for the α-subunit and to coordinate its delivery to the PM (Geering 2008; Tokhtaeva et al. 2009). In addition, the presence of the β-subunit is required for the activity of the enzyme. Particularly, reduction of disulfide bridges in the β-subunit results in inactivation of the enzyme and loss of cation occlusion (Lutsenko and Kaplan 1993). Furthermore, different isoforms of the Na,K-ATPase β-subunit are known to perform “fine tuning” of the ion-transporting function (Crambert et al. 2000; Geering 2008; Stanley et al. 2015). Moreover, the Na,K-ATPase β-subunit participates in maintaining epithelial cell polarity and cell adhesion by direct binding of the extracellular domains of the β-subunits located in neighboring cells (Cereijido et al. 2012; Vagin et al. 2012). Both proteinaceous sequences and N-glycans are involved in this β:β interactions (Tokhtaeva et al. 2011). Disruption of the Na,K-ATPase-β:β interactions either by altering the N-glycosylation site sequences or by adding competing Na,K-ATPase-β-specific antibodies prevents formation of junctional complexes that leads to an elevated paracellular permeability (Tokhtaeva et al. 2012; Vagin et al. 2008). Moreover, the Na,K-ATPase β-subunit is involved in the regulation of desmosomes and actin dynamics (Geering 2008; Rajasekaran et al. 2005, 2001b).

The activity and PM expression of the Na,K-ATPase α:β-complex is modulated by a third regulatory γ-subunit, belonging to the FXYD protein family (Sweadner and Rael 2000). As reviewed previously, seven different FXYD proteins that share a PFxYD motif in their N terminus have been detected in mammalian cells (Clausen et al. 2017; Geering 2006). The expression and function of FXYD proteins are tissue-specific and most of them act as inhibitors of Na,K-ATPase activity by either lowering the affinity of the transported ions to the enzyme or by modifying the pumping activity of the Na,K-ATPase (Garty and Karlish 2006; Geering 2005). In addition, it has been demonstrated that FXYD5 impairs cellular adhesion by disruption of cellular junctions between Na,K-ATPase β-subunits in epithelial cells (Tokhtaeva et al. 2016) and increase the surface expression of the tumor necrosis factor (TNF)-α receptor; thus, playing a pro-inflammatory role (Lubarski-Gotliv et al. 2016).

Generally, short-term regulation of the Na,K-ATPase involves mechanisms that affect the PM abundance of the enzyme and/or the function of PM-localized Na,K-ATPase molecules. Various stimuli trigger signaling pathways that mediate retrieval of the assembled Na,K-ATPase from the cellular PM via endocytosis. These processes are well characterized in the alveolar epithelium in the context of hypoxia and hypercapnia and are mediated by second messengers and intracellular kinases. Both hypoxia and hypercapnia lead to a marked and transient elevation of intracellular Ca^2+^ concentrations that subsequently stimulates AMP-activated protein kinase (AMPK) (Gusarova et al. 2009, 2011; Vadasz et al. 2008). In the setting of hypercapnia, stimulation of AMPK also requires prior activation of extracellular signal-regulated kinase (ERK)1/2 (Welch et al. 2010). In turn, AMPK drives translocation of protein kinase C (PKC)-ζ to the PM, where the kinase directly phosphorylates the Na,K-ATPase α-subunit at Ser^18^, thereby initiating internalization of the Na,K-ATPase from the cell surface (Gusarova et al. 2009, 2011; Vadasz et al. 2008). AMPK and PKC-ζ also activate the c-Jun N-terminal kinase (JNK)1/2 that subsequently promotes actin reorganization via phosphorylation of the LIM domain-only 7b (LMO7b) protein resulting in endocytosis of the Na,K-ATPase (Dada et al. 2015; Vadasz et al. 2012a). Of note, stimulation of AMPK and reduction of Na,K-ATPase activity have also been demonstrated in the settings of influenza virus infection (Peteranderl et al. 2016). Interestingly, cAMP has also been shown to be involved in the short-term regulation of the Na,K-ATPase PM expression via stimulation of protein kinase A (PKA) activity by soluble adenylyl cyclase (sAC) and modifications of the actin cytoskeleton (Lecuona et al. 2013). The involvement of cAMP (Bertorello et al. 1999), AMPK (Lang and Foller 2014), PKA (Cheng et al. 1997; Poulsen et al. 2010) and Ca^2+^ signaling (Aperia et al. 2016, 2020) has also been clearly demonstrated in additional studies focusing on the regulation of Na,K-ATPase abundance at the PM and/or on activity of the enzyme.

Long-term regulation of the Na,K-ATPase is primarily mediated at the transcriptional level by altering mRNA levels of its subunits. Previous reports have shown that hypoxia, glucocorticoids, insulin, progesterone, thyroid hormones, transforming growth factor-β and fibroblast growth factors alter transcription of the Na,K-ATPase (Clerici and Matthay 2000; Devarajan and Benz 2000; Li and Langhans 2015). Interestingly, transcriptional regulation of the transporter often has a positive feedback loop, where upregulation of a single Na,K-ATPase subunit leads to stimulation of the transcription and translation of another one. For example, overexpression of the Na,K-ATPase β-subunit results in upregulation of the α-subunit of the enzyme (Azzam et al. 2002; Rajasekaran et al. 2004).

The function of the Na,K-ATPase critically depends on its tissue expression and localization. In the lungs, the Na,K-ATPase drives vectorial transport of Na^+^ and in concerted action with epithelial sodium channels (ENaC), thereby generating an osmotic gradient that drives passive movement of water through epithelial monolayers; thus, maintaining a minimal epithelial lining fluid volume (Vadasz et al. 2007). Apart from the lungs, various other tissues and organs utilize the Na,K-ATPase-driven Na^+^ transport to maintain their functions. For example, the transporter participates in heart muscle metabolism (Shattock et al. 2015), drives vascular and endothelial functions, regulates reuptake of neurotransmitters in neurons (Mohan et al. 2019), controls electrolyte balance, blood pH and pressure and modulates reabsorption of amino acids and glucose in kidneys (Clausen et al. 2017; Matsuzaki et al. 2007). Due to its numerous functions and ubiquitous expression, dysfunction of the Na,K-ATPase has been linked to several pathological conditions and diseases. It is well documented that dysregulation of the Na,K-ATPase function leads among others to lung edema formation and persistence thus leading to progression of acute respiratory failure (Matthay et al. 2019; Mutlu and Sznajder 2005; Sznajder et al. 2002; Vadasz et al. 2007). In line with this notion, it has recently been demonstrated that knockout of the Na,K-ATPase β-subunit is associated with reduced alveolar fluid clearance in murine lungs in vivo (Flodby et al. 2016). Dysregulation of the Na,K-ATPase also leads to heart muscle hypertrophy and manifestation of chronic heart failure (Shattock et al. 2015), diabetes (Vague et al. 2004) and obesity (Obradovic et al. 2013). The dysfunction of the Na,K-ATPase during these conditions might be a consequence of expedited retrieval of the transporter from the PM or a reduced delivery of the enzyme to the cell surface. To what extent these mechanisms may involve altered protein folding and assembly of the Na,K-ATPase subunits in the ER is currently a topic of intense research in our laboratories.

## Protein Maturation, ER Stress and Unfolded Protein Response

Approximately one-third of the cellular proteome, most of the secreted and all PM proteins, including the Na,K-ATPase subunits, undergo co- and post-translational maturation in the ER (Brodsky and Skach 2011). The ER is a specific cellular organelle that coordinates co- and post-translational protein modifications, such as N-linked glycosylation, reduction of disulfide bonds, cleavage of sequences, proline isomerization and addition of glycophosphatidylinositol-anchors (Ellgaard and Helenius 2003; Ellgaard et al. 2018). In order to perform these processes, the ER requires high Ca^2+^ levels, an oxidizing environment and high levels of ATP (Jager et al. 2012). Furthermore, proper protein folding is tightly coordinated by specific ER-resident chaperones, mostly by binding immunoglobulin protein (BiP, also known as GRP78), which can facilitate folding of all proteins and by calnexin or calreticulin that facilitate folding of glycoproteins. In addition, protein oxidation reactions are controlled by reductases, such as DnaJ homolog subfamily B member (ERdj3-6), protein disulfide-isomerase A3 (ERp57) or endoplasmic reticulum oxidoreductase-1α (ERo1α) (Halperin et al. 2014). Addition of a 14-oligosaccharide core (Glc3Man9GlcNAc2) from a phosphate precursor or lipid carrier to the N-glycosylation site of the nascent folding peptide by glycosyltransferases results in formation of a monoglycosylated glycoprotein, thereby enhancing interaction of the folding protein with calnexin and calreticulin, thus activating the protein maturation cycle in the ER (Aebi 2013; Ellgaard and Helenius 2003).

The calnexin/calreticulin cycle is a central regulator of folding, quality control and degradation of newly-made glycoproteins (Wang and Kaufman 2016). Diverse physiological and pathological stimuli may affect protein structure, the folding environment of and the chaperone activity in the ER, thus inducing accumulation of misfolded or unfolded proteins in the ER and subsequent ER stress (Wang and Kaufman 2016). In particular, a decrease in calcium levels, changes in redox conditions or a reduction of ATP levels in the ER have been shown to impair ER homeostasis and induce ER stress (Han et al. 2013; Sano and Reed 2013). In response to ER stress, the unfolded protein response (UPR) is activated. Up to now, three main UPR pathways, termed by ER-localized receptors, namely inositol-requiring enzyme 1 (IRE1), protein kinase RNA-activated (PKR)-like ER kinase (PERK) and activating transcription factor-6 (ATF6) have been characterized. A dissociation of BiP from the ER-receptors, due to attachment of the chaperone to accumulated unfolded or misfolded proteins, leads to autophosphorylation and activation of the receptors. The UPR pathways initially aim to restore ER homeostasis by decreasing protein synthesis, by enhancing chaperone activity and by activating ER-associated degradation (ERAD) of the unfolded or misfolded proteins. However, if the UPR fails to restore ER homeostasis, a maladaptive response is activated that results in cellular death via apoptotic pathways (Almanza et al. 2019; Hwang and Qi 2018).

## Maturation of the Na,K-ATPase in the ER

**Fig. 1:**
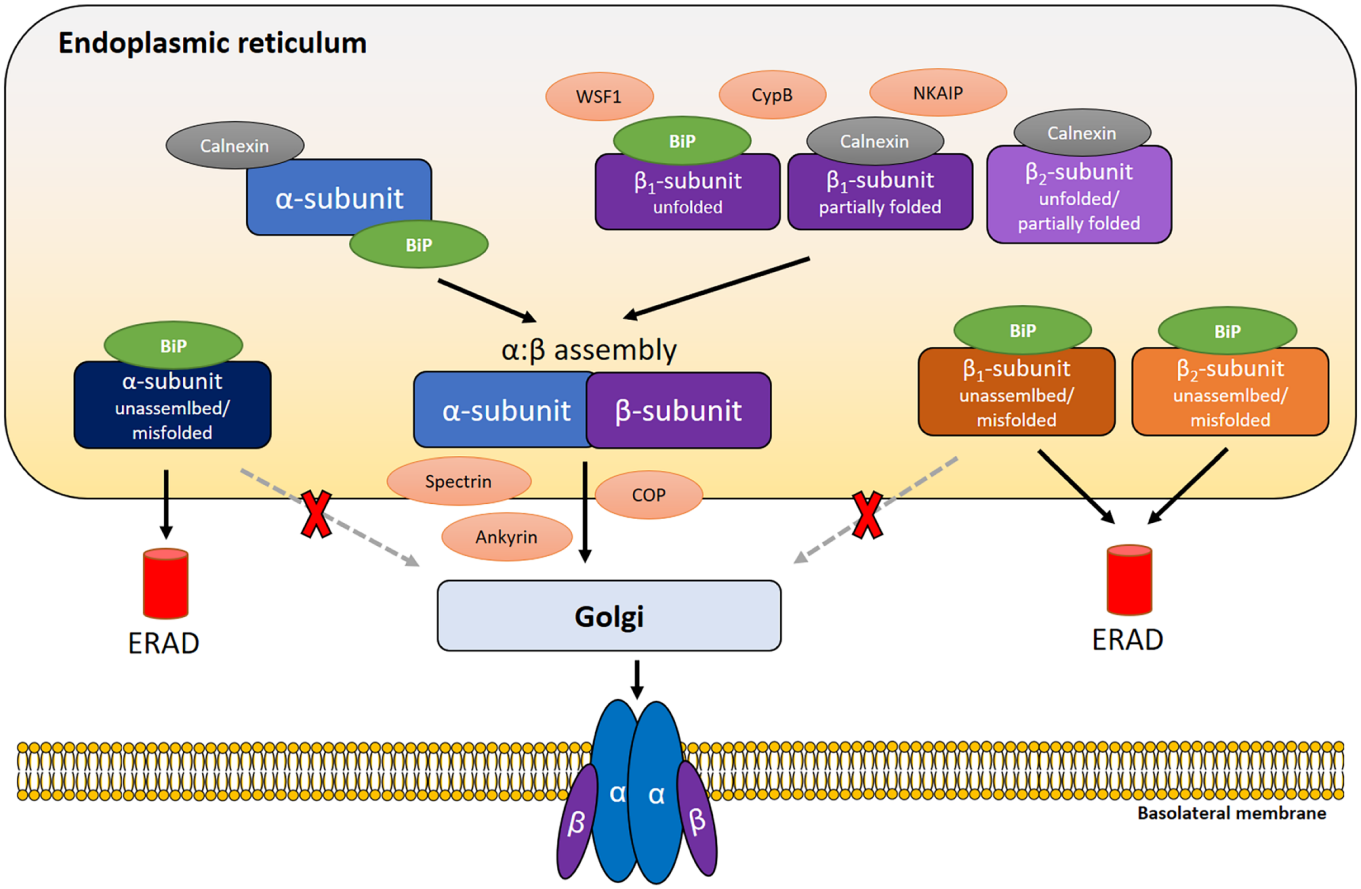
Schematic representation of Na,K-ATPase folding in the endoplasmic reticulum. The Na,K-ATPase α- and β-subunits are present in the ER in different physiological or pathophysiological states, including properly folded, unfolded or misfolded subunits of the enzyme and assembled complexes. Calnexin and BiP are ER chaperones that assist the folding of the Na,K-ATPase subunits with isoform specificity and preferential binding properties. Additionally, wolframin (WSF1), cyclophilin B (CypB) and Na,K-ATPase interacting protein (NKAIP) associate with the subunits of the Na,K-ATPase in the ER with divers functions. Only assembled Na,K-ATPase α:β-complexes can exit the ER and transferred to Golgi, whereas unassembled or misfolded subunits are retained with the assistance of BiP and targeted for endoplasmic reticulum-associated degradation (ERAD). Spectrin, ankyrin and coated proteins (COP) assist trafficking of assembled Na,K-ATPase α:β-complexes from the ER to the Golgi apparatus

The ER plays a crucial role in the folding of the Na,K-ATPase prior to delivery of the enzyme to the PM. Our current understanding on the maturation processes of the transporter in the ER is depicted in Fig. 1. Both co- and post-translational folding of the Na,K-ATPase subunits are coordinated by ER-resident chaperons, such as BiP and calnexin (Beggah et al. 1996; Beggah and Geering 1997; Tokhtaeva et al. 2010b). Previous reports suggest that both the unassembled Na,K-ATPase α- and β-subunits interact with BiP (Beggah et al. 1996). Furthermore, it has been shown that BiP plays a major role in the maturation of the Na,K-ATPase β-subunit. Folded Na,K-ATPase β_1_- or β_2_-subunits have several maturation states in the ER and either bind to α-subunits of the Na,K-ATPase and are subsequently guided for further maturation in the Golgi or bind to BiP and are subsequently retained in the ER and degraded by the ERAD machinery (Tokhtaeva et al. 2010b). In addition, inhibition of glycan-calnexin interactions or deletion of N-glycosylation sites of the Na,K-ATPase-β_1_ increases ER retention of the enzyme and its binding to BiP but does not affect assembly with Na,K-ATPase-α_1_ (Tokhtaeva et al. 2010b). In contrast, Na,K-ATPase-β_2_ is co-translationally bound to calnexin and disruption of this binding or decrease in N-glycosylation prevents the α:β-complex formation. Moreover, deletions or mutations in the Na,K-ATPase α_1_:β_1_ or α_1_:β_2_ interacting regions impair Na,K-ATPase α:β-complex formation, increase interaction of unassembled Na,K-ATPase subunits with BiP, ER retention and subsequent degradation (Tokhtaeva et al. 2009, 2010b). Interestingly, the degree of ER retention of the Na,K-ATPase subunits appears to be cell specific and is presumably dependent on different ratios of newly synthetized α- and β-subunits in different cell types (Marcus et al. 2020).

Most importantly, it has been demonstrated that the individual unassembled subunits of the Na,K-ATPase cannot leave the ER (Tokhtaeva et al. 2009). The above-described chaperone-assisted processes in the maturation of the Na,K-ATPase ensure that only intact Na,K-ATPase-α:β heterodimers with a proper stoichiometric ratio of 1:1 can be exported to the Golgi for subsequent maturation (Tokhtaeva et al. 2009). During trafficking from the ER to the Golgi, the Na,K-ATPase interacts with coat proteins (COP), which form specific vesicles required for retrograde transport from the Golgi to the ER or anterograde movement through the Golgi cisternae. Previous studies have reported that although Na,K-ATPase α-subunit may directly interact with COP, only α-subunits assembled with the β-subunits are able to translocate into the Golgi compartments. The unassembled α-subunits are retained in the ER and degraded via a proteasome-dependent mechanism (Morton et al. 2010). This finding underlines the importance of the Na,K-ATPase α:β-complex formation in the trafficking of the transporter to the PM.

Glycosylation also plays an important role in the maturation and trafficking of the Na,K-ATPase. All three isoforms of the β-subunit are N-glycosylated, whereas some FXYD proteins are O-glycosylated. During the initial step of Na,K-ATPase-β glycosylation in the ER, and oligosaccharide core is added, which results in a shift of the molecular weight of the protein by formation of high mannose type of N-glycans (Tokhtaeva et al. 2010b). During subsequent maturation of the Na,K-ATPase in the ER, N-glycans are added to the β-subunit by assistance of ER- and Golgi-resident glycosidases and glycosyltransferases. This leads to the formation of hybrid- or complex-type N-glycans (Vagin et al. 2012). Of note, N-glycans are not essential for Na,K-ATPase α_1_:β_1_ assembly, insertion of the enzyme to the PM or its activity (Vagin et al. 2012). In contrast, N-glycans are critical for the formation, maintenance and regulation of epithelial junctions (Vagin et al. 2006, 2008). On the other hand, removal of the N-glycosylation sites is associated with increased susceptibility to degradation of the Na,K-ATPase β-subunit in the ER (Laughery et al. 2003). In contrast to the β_1_-subunit, the presence of N-glycans plays an important role in the assembly of the α-subunit with the β_2_-subunit of the Na,K-ATPase (Tokhtaeva et al. 2010a). Moreover, disulfide bonds in the Na,K-ATPase β-subunit are not necessary for the assembly of the heterodimer, however, are crucial for ER exit and PM targeting of the transporter, since truncated versions or mutants of the Na,K-ATPase-β with disulfide bridge disruptions are able to assemble with the α-subunit but are retained in the ER (Laughery et al. 2003).

Apart from ER-resident chaperones, other proteins may participate in the maturation of the Na,K-ATPase. For example, the Na,K-ATPase-β_1_ has been shown to interact with wolframin (WSF1), an ER-localized protein, as initially identified in a yeast two-hybrid screening assay. Of note, WSF1 mutants or a knockdown of WSF1 reduce the expression of both Na,K-ATPase-β_1_- and -α_1_ at the PM, suggesting that WSF1 may be required for maturation of the Na,K-ATPase in the ER (Zatyka et al. 2008). Another report showed an interaction between the Na,K-ATPase β_1_-subunit and cyclophilin B (CypB). CypB is an enzyme from the cyclophilin family, members of which are known to have peptidyl prolyl *cis–trans* isomerase activity, thus possessing chaperon activity and are involved in the folding and repair of proteins. CypB expresses an ER-directed signal sequence and participates in protein maturation in the ER. Interestingly, silencing CypB results in an increase of ER abundance of Na,K-ATPase-α and -β as well as decreased transporter activity (Sune et al. 2010). Although these results suggest that CypB might regulate maturation of the Na,K-ATPase in the ER, the exact molecular mechanisms remain to be determined. Another protein that has been found to interact with the Na,K-ATPase β-subunit in the ER and in lysosomes is Na,K-ATPase interacting protein (NKIP), an endogenous suppressor of the activity of the enzyme with a currently unknown role in ER processing of the Na,K-ATPase (Pratscher et al. 2008). Furthermore, it has been shown that the Golgi-localized spectrin-ankyrin skeleton is also required for Na,K-ATPase trafficking from the ER to the Golgi. Interestingly, genetic modification of spectrin blocks transport of both Na,K-ATPase-α and -β from the ER, but does not interfere with the formation of Golgi stacks, the distribution of COP or trafficking and surface expression of E-cadherin, suggesting selectivity for the Na,K-ATPase (Devarajan et al. 1997). In line with this notion, blocking the ankyrin-binding sequence of the Na,K-ATPase-α_1_ inhibits the ER to Golgi transport of the subunit (Stabach et al. 2008). Interestingly, ankyrin and Na,K-ATPase have been found to interact with the ER-localized inositol trisphosphate receptor (InsP3R), thus directly participating in the regulation of luminal Ca^2+^ concentration of the ER (Cook et al. 2012; Turner et al. 2016). Whether there is a feedback loop among maturation of the Na,K-ATPase in the ER, trafficking of the enzyme from the ER to the Golgi and ER Ca^2+^ concentrations, is currently unknown and warrants further investigation.

## ER Dysfunction-Related Impaired Maturation of the Na,K-ATPase in Disease States

Recent studies reported that changes in the ER microenvironment, such as a decrease in Ca^2+^ levels, ATP or alterations in the oxidative environment of the ER, lead to protein misfolding or unfolding, induce ER stress and activate UPR pathways (Araki and Nagata 2011; Wang and Kaufman 2016). Decreased PM expression and function of the Na,K-ATPase have been shown in various pathological lung, heart, renal and neurological conditions and diseases (Bonilla et al. 1991; de Lores Arnaiz and Ordieres 2014; Fekete et al. 2008; Matsuzaki et al. 2007; Shattock et al. 2015; Vadasz et al. 2007).

It has been reported that cadmium (Cd^2+^), which promotes oxidative stress and lipid peroxidation, induces ER retention of the Na,K-ATPase β_1_ and β_2_ subunits. After Cd^2+^ treatment, both subunits are retained in ER in a dose-dependent manner, whereas retention of the β_2_-subunit appears to be more prominent. Additionally, Cd^2+^ treatment results in a marked and selective increase in BiP levels as opposed to calnexin, which in turn rescues maturation of the Na,K-ATPase β_1_- but not of the β_2_-subunit. As the Na,K-ATPase α_1_:β_1_-complex plays a pivotal role in maintaining cellular membrane potential and thus in cellular survival, the resistance of the Na,K-ATPase-β_1_ to ER stress may serve as an adaptive mechanism during dysfunction of the ER potentially contributing to cellular resilience (Tokhtaeva et al. 2010b).

Various acute and chronic lung diseases are associated with an elevation in carbon dioxide (CO_2_) levels in blood and tissues, a condition termed hypercapnia (Vadasz et al. 2012b). It has been previously shown that an acute exposure of lung alveolar epithelial cells to elevated CO_2_ concentrations results in downregulation of Na,K-ATPase function by increased trafficking of the enzyme from the PM into intracellular compartments (Dada et al. 2015; Lecuona et al. 2013; Vadasz et al. 2008; Welch et al. 2010). Additionally, it has recently been shown that sustained hypercapnia impairs maturation of the transporter in the ER. In particular, elevated CO_2_ levels promote ER retention of the Na,K-ATPase β-subunit in lung epithelial cells, thus decreasing PM abundance and activity of the enzyme (Kryvenko et al. 2020). These negative effects of CO_2_ on the Na,K-ATPase are driven by alterations of the oxidizing environment and direct carbonylation of the Na,K-ATPase β-subunit in the ER during hypercapnia, which impairs α:β-complex formation and subsequent trafficking to the PM. Interestingly, both BiP and calnexin interact with Na,K-ATPase-β_1_ upon CO_2_ exposure; however, levels of BiP remain unchanged, suggesting that as opposed to Cd^2+^ treatment, upon hypercapnia folding of Na,K-ATPase-β_1_ is not rescued (Kryvenko et al. 2020). Another recent study suggests that a decrease of the expression of the Na,K-ATPase β_1_-subunit drives ER stress and promotes a fibrotic phenotype in alveolar epithelial cells. Interestingly, specific knockdown of Na,K-ATPase-β_1_ leads to increased expression of BiP, fibronectin and α-smooth muscle actin, thus activating ER stress and fibrotic signaling pathways, suggesting that mechanisms that impair expression of the Na,K-ATPase β_1_-subunit may contribute to development of lung fibrosis (Li et al. 2019).

Another recent report shows that infection of gastric epithelial cells with *Helicobacter pylori* impairs chaperone-assisted maturation of newly synthetized Na,K-ATPase molecules prior to trafficking of the enzyme to the PM (Marcus et al. 2020). An infection with *H. pylori* prevents association of BiP with both the α- and the β-subunits of the Na,K-ATPase in the ER, thus, causing defective folding and subsequent ubiquitination and proteasomal degradation of the Na,K-ATPase subunits, thereby inhibiting formation of α_1_:β_1_ heterodimers. Interestingly, this impairment of Na,K-ATPase maturation in the ER is not associated with ER stress, increased total levels of BiP or prevention of BiP-assisted chaperone folding of other proteins. However, overexpression of BiP, inhibition of protein synthesis or blocking of proteasomal degradation partially rescue maturation of the Na,K-ATPase upon *H. pylori* infection. A decrease in Na,K-ATPase levels has been observed in chronically infected children and in gastric epithelia of gerbils in vivo (Marcus et al. 2020). Although a direct link between the decreased levels of the Na,K-ATPase and the impairment of the barrier function of gastric epithelia by *H. pylori* is to be explored, the expected physiologic consequence is a decrease in the inward Na^+^ gradient with intracellular accumulation of Na^+^, resulting in the impairment of ion homeostasis and nutrient uptake, cell swelling, damage to cell junctions and subsequent gastric injury. Along with these expectations, imaging studies demonstrated accumulation of *H. pylori* clusters at the junctions between significantly swollen cells in the infected epithelium from human biopsy samples (Fiocca et al. 1987). Further understanding of *H. pylori*-induced Na,K-ATPase degradation will provide insights for protection against advanced disease and may provide novel therapeutic targets in the context of gastric epithelial injury.

Mutations in the catalytic α-subunit of the Na,K-ATPase have been shown to be involved in the pathogenesis of the several neurological disorders, such as epilepsy, cerebellar ataxia, familial hemiplegic migraine and axonal Charcot–Marie–Tooth neuropathy (Dard et al. 2015; Friedrich et al. 2016; Lassuthova et al. 2018). However, the clinical manifestations of some of these disorders are not a consequence of impaired PM function of the Na,K-ATPase but rather the altered trafficking of the transporter (Arystarkhova et al. 2019). For example, a recent report demonstrates that mutations of the Na,K-ATPase α_3_-isoform, which have been found in severe cases of early infantile epileptic encephalopathy, result in decreased biosynthesis and trafficking of the transporter, but do not alter activity of the enzyme. Instead, these mutations induced ER retention of the β_1_-subunit and subsequent activation of UPR via increased phosphorylation of eukaryotic initiation factor 2 α and leading to ERAD of the α_3_-subunit. In line with this notion, treatment with the chemical chaperone 4-PBA appears to attenuate the effects of the Na,K-ATPase α_3_ mutations, rescuing the ER-retained β_1_-subunit and improving cellular morphology (Arystarkhova et al. 2020).

It has previously been shown that in kidney biopsies from patients with autosomal dominant polycystic kidney disease, the Na,K-ATPase β_1_-subunit is predominantly expressed in the cytoplasm and the ER, as opposed to kidney biopsies from patients without the disease in which the α_1_- and β_1_-subunits of the transporter are localized at the basolateral membrane of renal tubules. Interestingly, ER retention of the β_1_-subunit is associated with a compensatory increase of the β_2_-subunit and apical, but not basolateral, expression of the Na,K-ATPase α_1_:β_2_-complex (Wilson et al. 2000). In contrast, this mislocation was not observed in animal models of autosomal dominant polycystic kidney disease (Kawa et al. 1994; Takahashi et al. 1997; Thomson et al. 2003). Thus, mistargeting of the Na,K-ATPase in the setting of this disease remains controversial (Zatti et al. 2005). Interestingly, a recent study observes aberrant apical expression of the Na,K-ATPase upon influenza A virus infection of lung epithelial cells both in vitro and in vivo (Peteranderl et al. 2019). However, the exact mechanism of this phenomenon remains unknown. Of note, various studies also suggest an aberrant localization of the Na,K-ATPase secondary to an infection with severe acute respiratory syndrome coronavirus 2 (SARS-CoV-2), which may contribute to alveolar epithelial barrier dysfunction, persistence of pulmonary edema and deterioration of patients with coronavirus disease 2019 (COVID-19)-associated acute respiratory distress syndrome (Kryvenko and Vadasz 2021).

In conclusion, recent evidence suggests that the ER plays an essential role in co- and post-translational processing of Na,K-ATPase subunits and assembly of the α:β heterodimer complexes. The maturation of the Na,K-ATPase is a multifaceted, subunit-specific process that is negatively affected by alterations in ER homeostasis, causing misfolding of the proteins, exacerbating ER stress and may lead to UPR. As impaired maturation of the Na,K-ATPase is associated with various disease states, better understanding of these mechanisms may lead to novel therapeutic means.
